# Decreased renal expression of PAQR5 is associated with the absence of a nephroprotective effect of progesterone in a rat UUO model

**DOI:** 10.1038/s41598-023-39848-2

**Published:** 2023-08-08

**Authors:** P. A. Abramicheva, D. S. Semenovich, L. D. Zorova, I. B. Pevzner, I. A. Sokolov, V. A. Popkov, E. P. Kazakov, D. B. Zorov, E. Y. Plotnikov

**Affiliations:** 1 A.N. Belozersky Institute of Physico-Chemical Biology, Lomonosov Moscow State University, Moscow, Russia 119234; 2 V.I. Kulakov National Medical Research Center of Obstetrics, Gynecology, and Perinatology, Moscow, Russia 117997; 3 Mendeleev University of Chemical Technology of Russia, Moscow, Russia 125047; 4 MSU Institute for Artificial Intelligence, Lomonosov Moscow State University, Moscow, Russia 119234

**Keywords:** Kidney, Steroid hormones, Obstructive nephropathy

## Abstract

Fibrosis is a severe complication of chronic kidney disease (CKD). Progesterone, like other sex hormones, plays an important role in renal physiology, but its role in CKD is poorly understood. We investigated progesterone effect on renal fibrosis progression in the rat model of unilateral ureteral obstruction (UUO). Female rats were exposed to UUO, ovariectomy and progesterone administration after UUO with ovariectomy. Expression of key fibrosis markers, proinflammatory cytokines, levels of membrane-bound (PAQR5) and nuclear (PGR) progesterone receptors, and matrix metalloproteinase (MMP) activity were analyzed in the obstructed and intact rat kidney. In all groups exposed to UUO, decreased *PAQR5* expression was observed in the obstructed kidney while in the contralateral kidney, it remained unaffected. We found increased mRNA levels for profibrotic *COL1A1*, *FN1*, *MMP2*, *TIMP1*, *TIMP2*, proinflammatory *IL1α*, *IL1β*, and *IL18*, as well as elevated α-SMA and MMP9 proteins, collagen deposition, and MMP2 activity in all UUO kidneys. Progesterone had slight or no effect on the change in these markers. Thus, we demonstrate for the first time diminished sensitivity of the kidney to progesterone associated with renal fibrosis due to a severe decrease in *PAQR5* expression that was accompanied by the lack of nephroprotection in a rat UUO model.

## Introduction

Chronic kidney disease (CKD) is characterized by gradual loss of kidney function and progression of fibrosis. Accumulation of extracellular matrix proteins is a prominent feature of tubulointerstitial fibrosis leading to end-stage renal disease^1^. Due to high morbidity and mortality associated with CKD, therapeutic approaches are being actively sought. Obstructive nephropathy caused by urinary calculi is one of the most common factors of CKD^2^. The experimental model of unilateral ureteral obstruction (UUO) used to cause renal fibrosis in rodents is believed to mimic human chronic obstructive nephropathy in an accelerated mode^3^.

CKD appears to be a gender-specific pathology: women are more likely to develop CKD, but when occurred, the rate of its progression is higher in men^4^. As with other kidney diseases, nephrolithiasis is 2–3 times more common in men than in women^5^. The incidence of renal hypertension and diabetic nephropathy is higher in men than in women of the same age before menopause^6,7^.

The role of progesterone in renal physiology is of particular interest. This steroid hormone is known to be involved in regulation of the female reproductive system and maintenance of pregnancy^8,9^, immune modulation^10,11^, and neuromodulation^12,13^. While estrogen treatment has a nephroprotective role in models of acute^14,15^ and chronic kidney failure^16–18^, the effects of progesterone remain unclear and controversial, particularly with regard to the progression of renal fibrosis. Progesterone signaling is mediated by membrane (mPR) and nuclear receptors (nPR). mPR gamma, or progestin and adipoQ receptor 5 (mPRγ/PAQR5) are expressed in the kidneys of various vertebrates^19^. Their distribution in the kidney and cellular localization has been shown^20^ but there is a significant lack of data on their renal function. Expression of nPRs (nPR-A and nPR-B) has also been identified in human kidney tissue and corresponding mammalian cell lines^8^. Although function of nPRs in the kidney remains unknown, there are a growing number of studies suggesting that nuclear receptors to various ligands play an important role in maintaining homeostasis and modulating renal disease progression^21,22^. The aim of this study was to assess the renal profile of PRs expression and to elucidate how renal progesterone sensitivity changes in obstructive nephropathy. We examined the intricate relationship between renal PRs expression and progesterone sensitivity, which may provide valuable insights for the development of new therapeutic approaches for the treatment of obstructive nephropathy.

## Results

### Morphology and relative kidney weight under obstructive nephropathy

**Figure 1:**
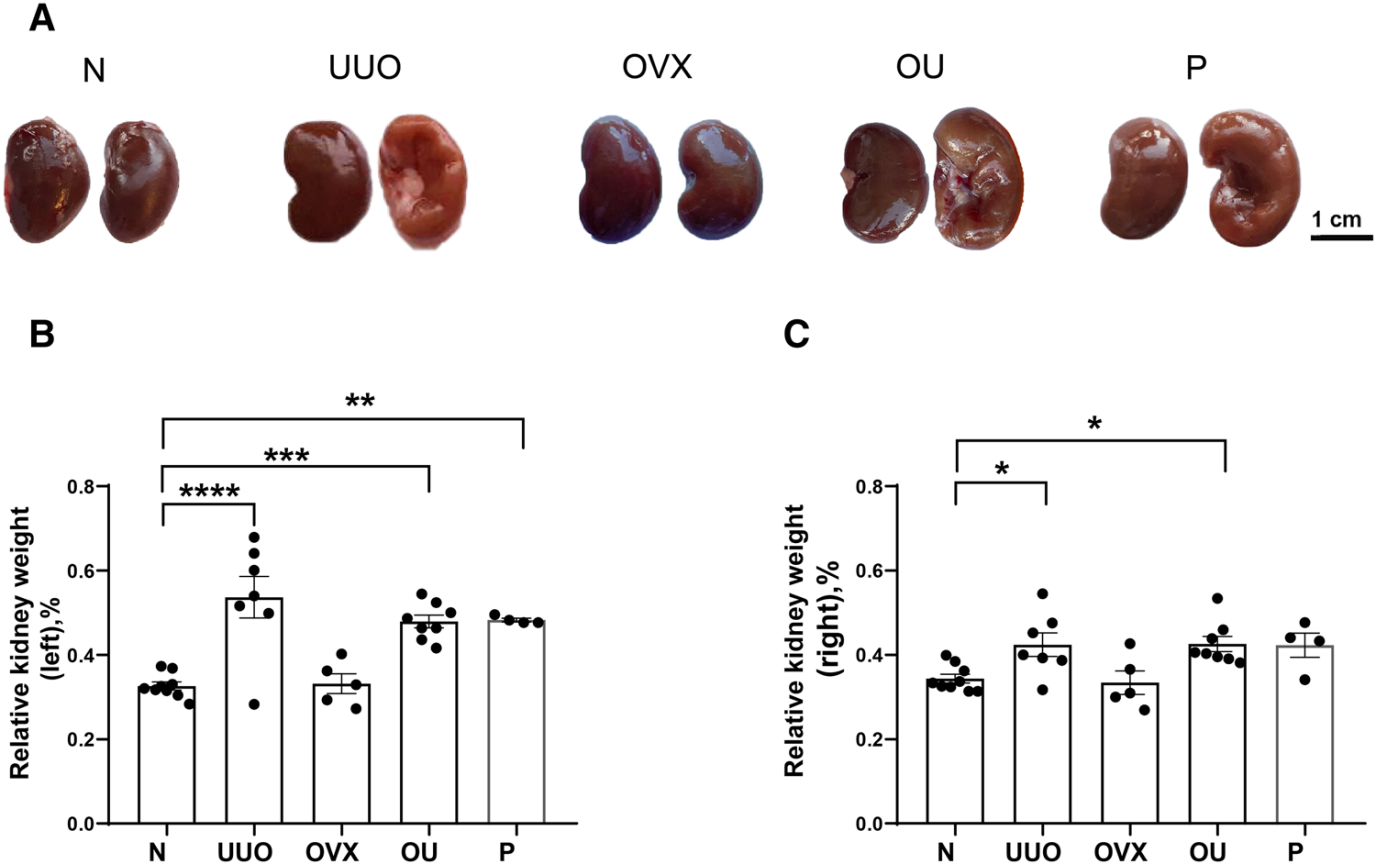
Changes in rat kidneys exposed to obstructive nephropathy. (**A**) Photographs of kidneys in all experimental groups. (**B**) Relative left kidney weight. (**C**) Relative right kidney weight. Asterisks indicate significance identified by Sidak’s multiple comparisons, one-way ANOVA. *P* values: *p < 0.05, **p < 0.01, ***p < 0.001, ****p < 0.0001, n = 4–9.

Renal morphology was analyzed for all experimental groups: intact rats (N); unilateral ureteral obstruction (UUO of the left kidney), bilateral ovariectomy (OVX); OVX followed by UUO (OU); OVX followed by UUO with progesterone administration (P). The left kidneys exposed to ureteral obstruction were severely altered: they were pale and showed hypertrophy caused by hydronephrosis (Fig. 1A). The relative kidney weight (kidney weight to body weight ratio) of both kidneys was increased in UUO, OU and P compared to N, indicating renal hypertrophy. There were no significant differences between the OU and P groups in either the left or the right kidney (Fig. 1B, C).

### *PAQR5* expression is downregulated in renal fibrosis

**Figure 2:**
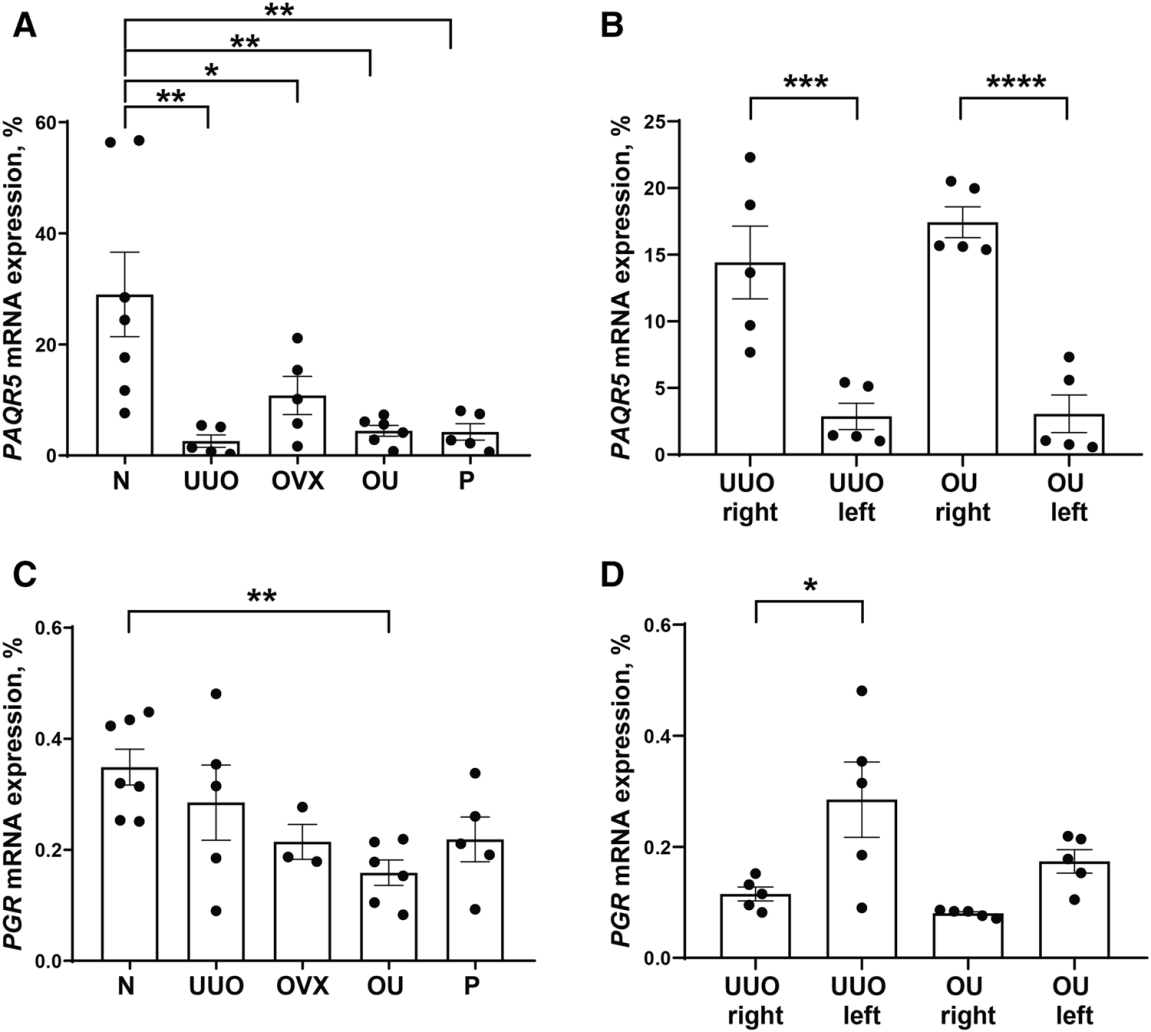
Expression of *PAQR5* in the damaged and intact kidney. *PAQR5* (**A**) and *PGR* (**C**) expression in the left (obstructed) kidney in all experimental groups. Comparison of left and right kidney expression of *PAQR5* (**B**) and *PGR* (**D**) in UUO and OU groups. *P* values: *p < 0.05, **p < 0.01, ***p < 0.001, ****p < 0.0001, n = 3–7.

RT-PCR revealed a significant decrease in *PAQR5* expression in the left kidney of groups U, OVX, OU, and P compared with intact rats (Fig. 2A), and protein levels of PAQR5 showed similar alterations (Fig. S1). However, in the right kidney of the same animals, mRNA expression of this receptor did not decrease in animals with ureteral obstruction, but remained at the level of expression in intact rats (Fig. 2B). Total expression of nPR-A and B (*PGR*) was reduced in the left kidney in OU group compared with control (Fig. 2C). In the right kidney, the expression of nPRs was decreased during ureteral obstruction compared to the left kidney (Fig. 2D).

### Fibrosis markers did not decrease under progesterone supplementation

**Figure 3:**
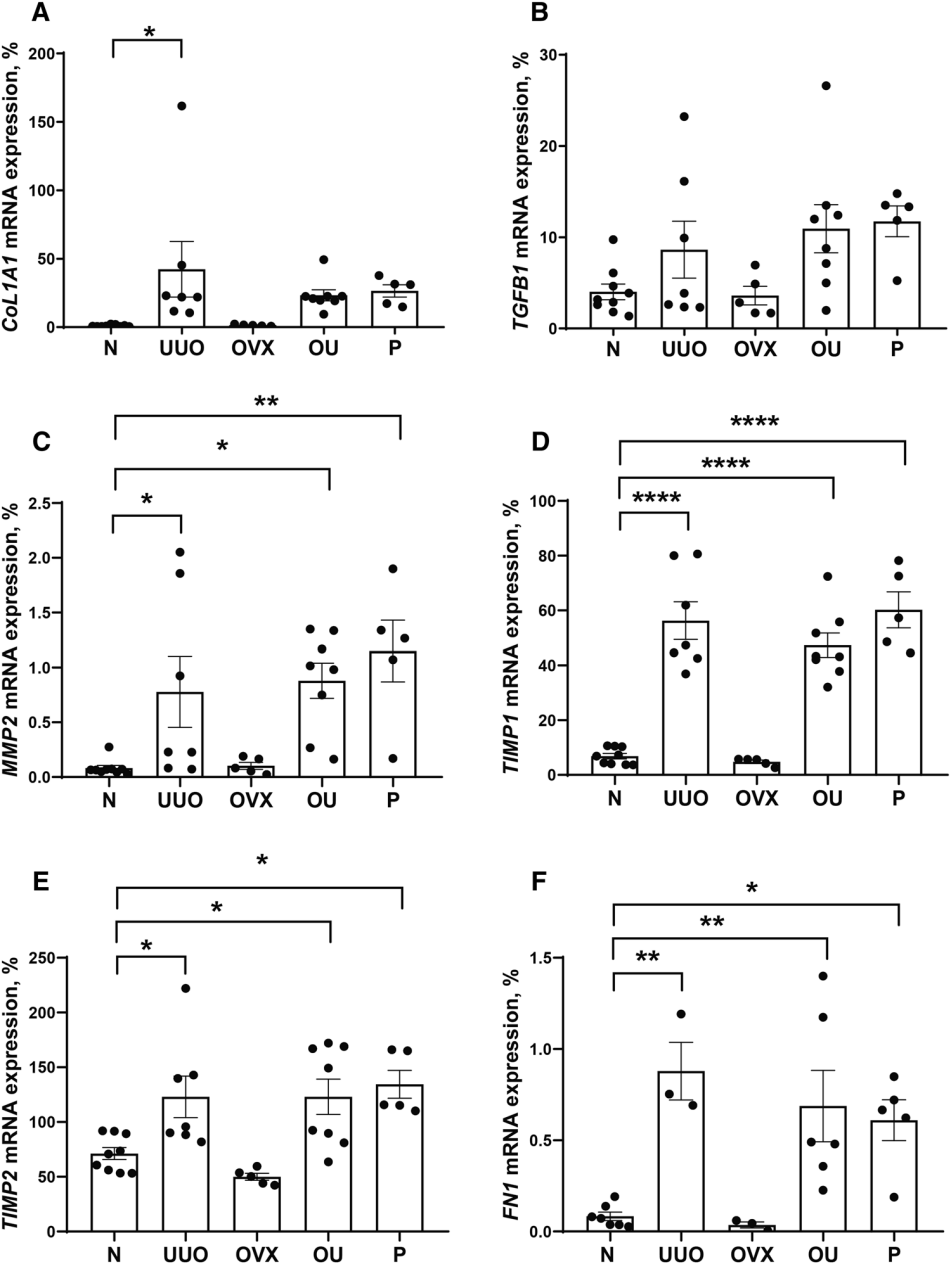
Expression of fibrosis markers in the left kidney. (**A**) *COL1A1*; (**B**) *TGFB1*; (**C**) *MMP2*; (**D**) *TIMP1* and (**E**) *TIMP2*; (**F**) *FN1*. Data is normalized to *RPLP0* and *HPRT*. *P* values: *p < 0.05, **p < 0.01, ***p < 0.001, ****p < 0.0001, n = 3–9.

**Figure 4:**
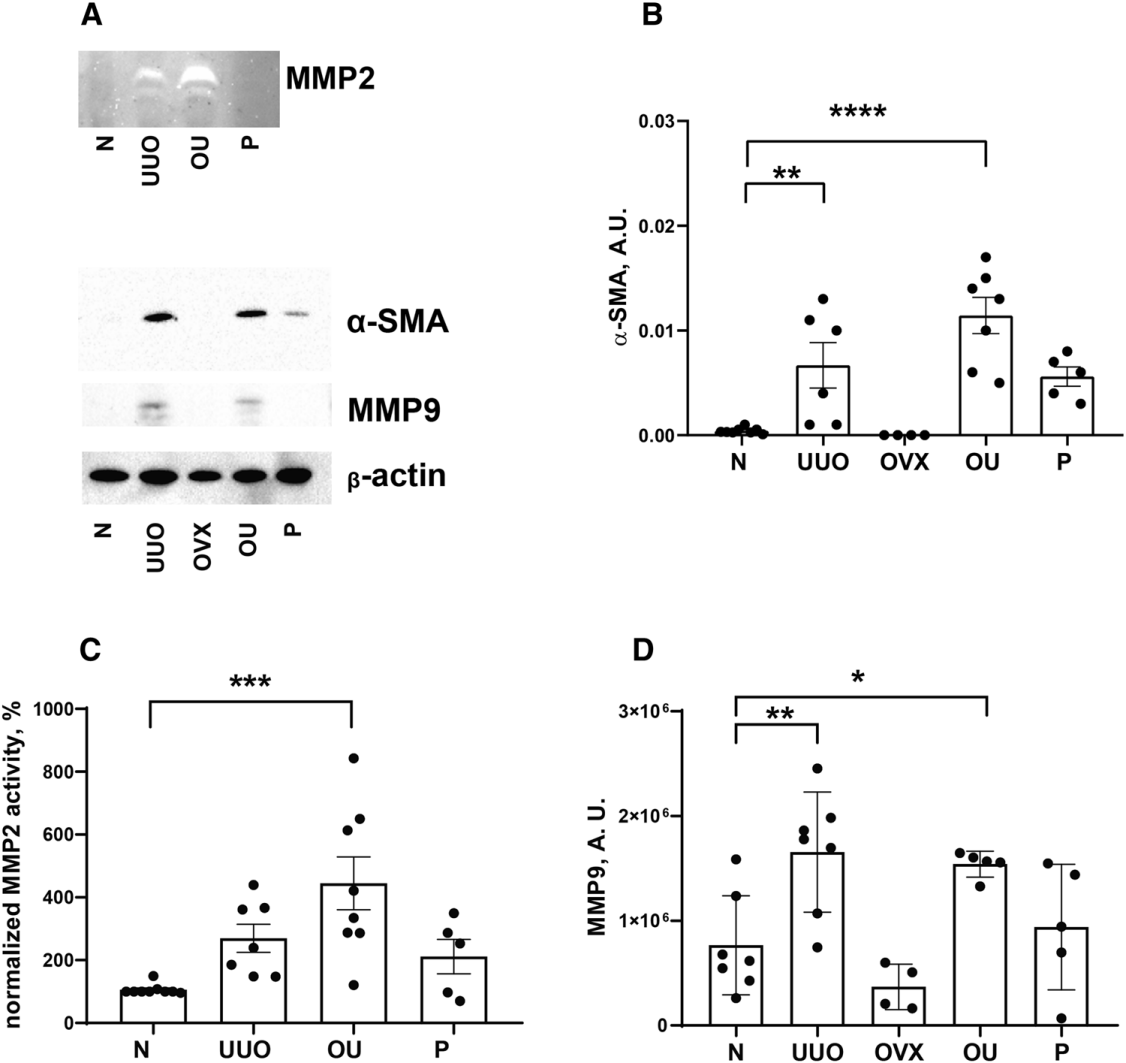
(**A**) Representative zymogram to MMP2 and blots to α-SMA and MMP9 with β-actin as a loading control. Densitometry for (**B**) α-SMA levels, (**C**) MMP2 activity, and (**D**) MMP9 levels in the left (UUO exposed) kidney. *P* values: *p < 0.05, **p < 0.01, ***p < 0.001, ****p < 0.0001, n = 4–9.

**Figure 5:**
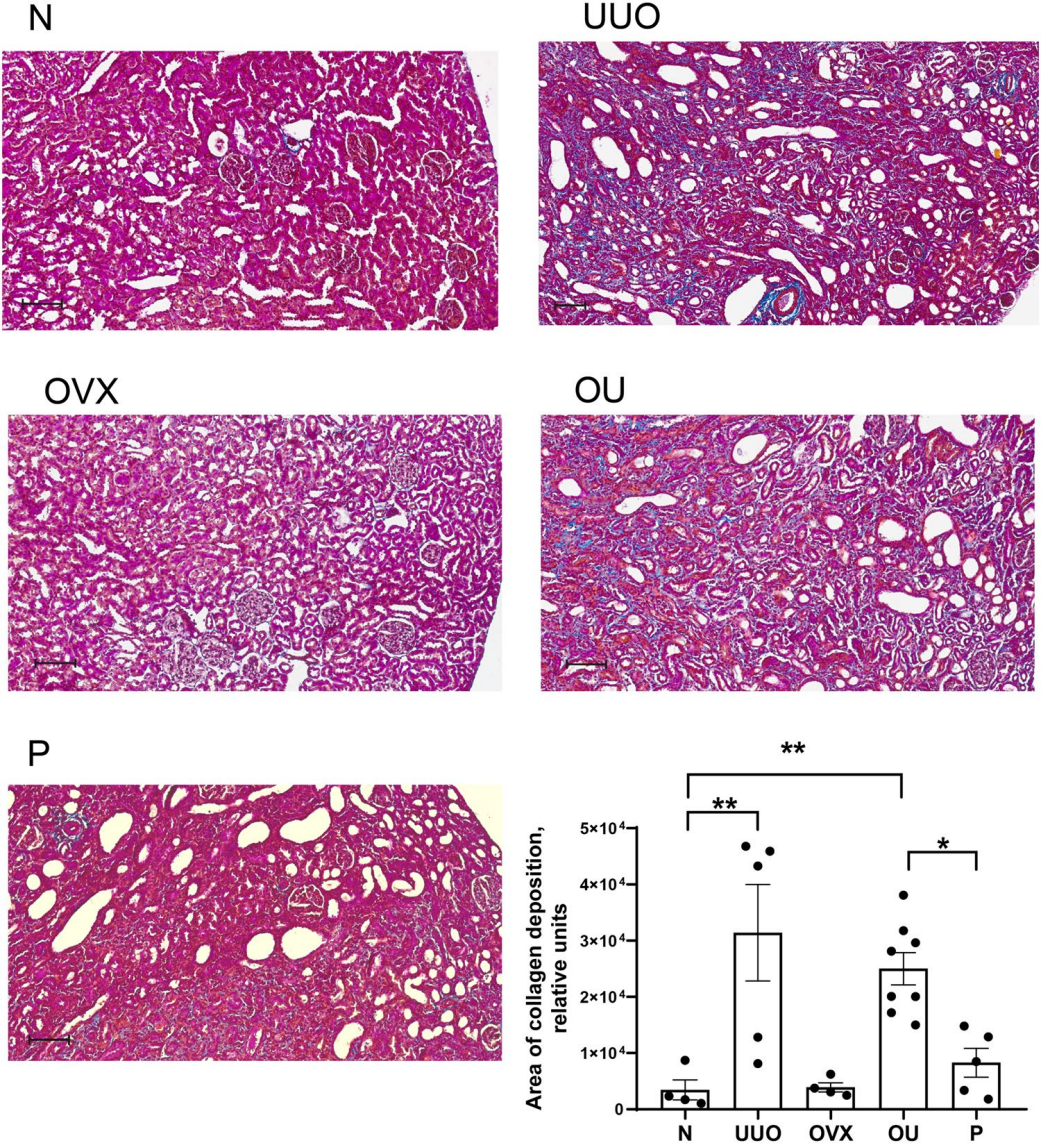
Representative Mallory staining of the section from the left kidney of animals exposed to different protocols. Bottom right is the statistics of staining. On each slice, ten images from the cortex were randomly selected for analysis. Bar, 100 μm. *P* values: *p < 0.5, **p < 0.01, n = 4–8.

An increase in the expression of fibrosis markers (collagen 1α (Fig. 3A), transforming growth factor β1 (Fig. 3B), matrix metalloproteinase 2 (Fig. 3C), tissue inhibitors of metalloproteinase 1 (Fig. 3D) and 2 (Fig. 3E), fibronectin (Fig. 3F)) was shown in the U, OU, and P groups compared with intact rats. Progesterone exposure did not affect renal fibrosis development caused by ureteral obstruction. Protein expression of α-smooth muscle actin (α-SMA), MMP2 activity and MMP9 protein levels changed similarly (Fig. 4B, C, and D, respectively). Mallory staining showed an increase in collagen deposition in the U and OU groups compared to intact rats, and decrease in the P group compared to OU (Fig. 5).

### Analysis of the immunomodulatory role of progesterone under obstructive nephropathy

**Figure 6:**
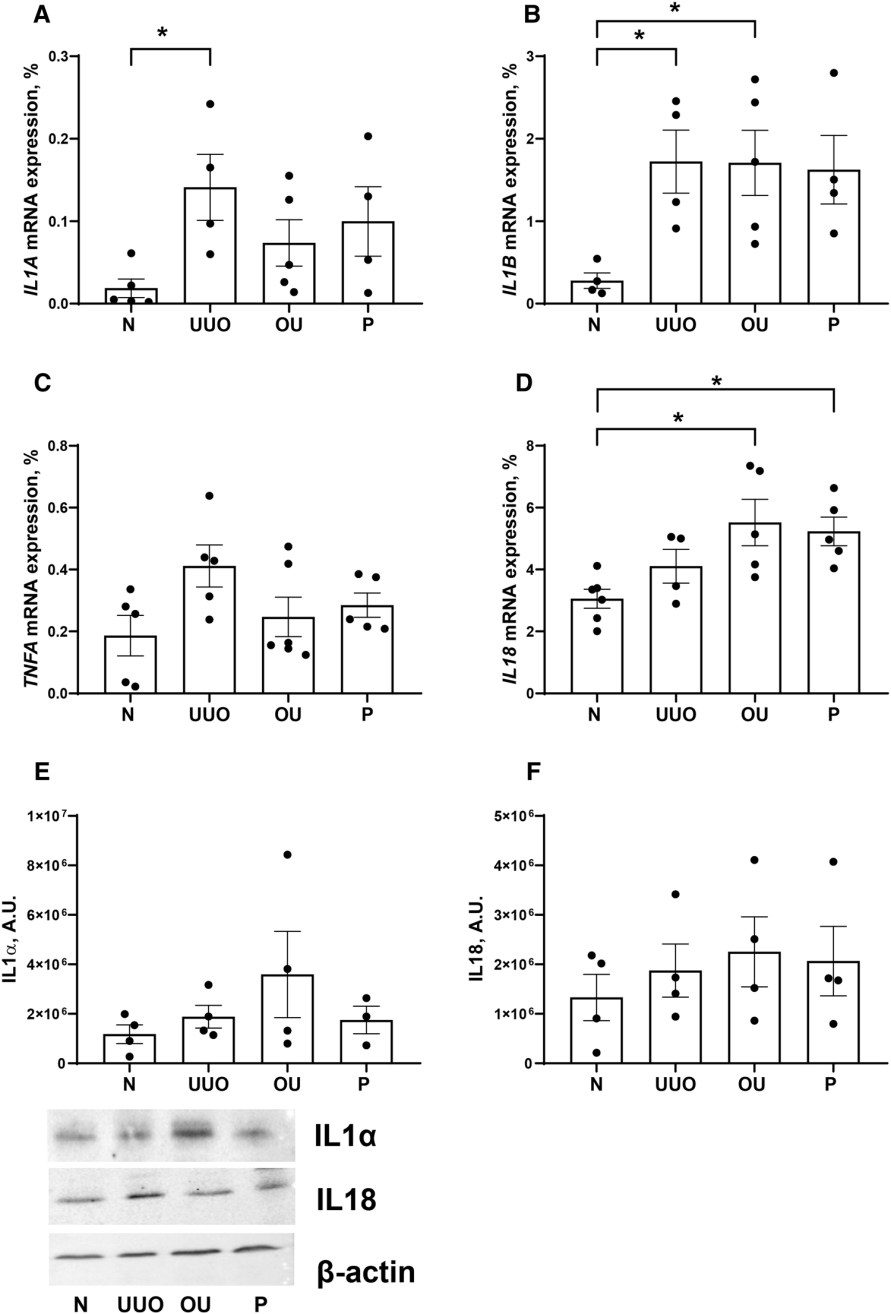
mRNA and protein expression of interleukins. (**A**) IL1α mRNA (**B**) IL1β mRNA. (**C**) TNFα mRNA. (**D**) IL18 mRNA. (**E**) IL1α protein. (**F**) IL18 protein. *P* values: *p < 0.05, n = 4–6.

mRNA expression of IL1α, IL1β and IL18 but not of TNFα increased in response to ureteral obstruction (Fig. 6A, B, D and C correspondingly). For mRNA, there were no significant differences between the OU and P groups, as well as levels of IL1α and IL18 proteins didn’t change either (Fig. 6E, F).

## Discussion

Despite the large body of works addressing the role of progesterone in non-reproductive tissues^23–26^, its role in the kidney has been poorly studied. Progesterone was long believed to exert its effects in the kidney by binding to high-affinity mineralocorticoid receptors^27–29^, until identification of progesterone receptors that are predominantly expressed in kidney tissue, namely the membrane receptors PAQR5 and the nuclear receptors nPR-A and nPR-B^20^. The renal system of progesterone-metabolizing enzymes has also been described^8^. PAQR5 is expressed almost throughout the entire nephron except in the glomerulus^20^ suggesting that progesterone realizes most of its non-genomic rapid effects via this receptor isoform. Currently, the following isoforms of PRs are known: nuclear (nPR-A and nPR-B), membrane (PAQR5-PAQR9) and progesterone receptor membrane components (PGRMC1, PGRMC2)^30,31^. Despite the diversity of isoforms and PRs-associated signaling cascades, most previous research on progesterone has focused on the classical progesterone-nPR signaling pathways^31^ and the role of PAQR5 still remains unexplored.

In this study, we found a dramatic decrease in *PAQR5* expression associated with renal fibrosis (Fig. 2) indicating a possible violation in progesterone signaling. Recently, it was shown that the expression of this mPR was significantly downregulated in tumor tissues from patients with renal cell carcinoma and inversely correlated with TGFβ1 expression^32,33^. In contrast, suppression of TGFβ1 signaling by pirfenidone resulted in increased PAQR5 expression in lung fibroblasts in idiopathic pulmonary fibrosis^34^. Moreover, renal carcinoma is usually associated with intratumoral fibrosis, and markers of fibrosis such as type I collagen and fibronectin promote tumor progression^35,36^, suggesting that kidney fibrosis contributes to decreased PAQR5 expression. Suppressed expression of the receptor leads to decreased hormone sensitivity of the target tissue, resulting in the absence of the effect of progesterone as observed in our work. Interestingly, there is evidence of a protective effect of progesterone in acute models of kidney injury in male rats^37^. It could be hypothesized that progesterone administration had some protective effect on the kidneys of male rats. Therefore, the distribution of progesterone receptors and the effects on the progression of renal fibrosis in male and female models of chronic kidney injury need to be further investigated.

We demonstrate that the expression of fibrosis markers did not decrease after progesterone administration to animals with UUO, either at the mRNA or protein level (Figs. 3, 4). A slight decrease in collagen deposition in response to progesterone administration was demonstrated by Mallory staining (Fig. 5). Similar effects were described for the diabetic nephropathy model, in which progesterone treatment attenuated the glomerulosclerosis index in the diabetic kidney^38^. To date, there are only a limited number of studies on the effects of progesterone on fibrosis, and only a few studies have shown the protective effect of progesterone on the kidneys. For example, in rats with hypertension induced by deoxycorticosterone acetate^39^, diabetic nephropathy^38^, lupus nephritis^40^, and ischemia reperfusion injury^37^ progesterone attenuated renal damage. It should be noted that, in most of the works dealing with the nephroprotective effects of progesterone, no attention has been paid to the assessment of the renal fibrosis progression and the analysis of PRs expression. Thus, we can assume that in the above models of kidney injury, fibrosis has not developed at all, or the hormone sensitivity of the tissue has not been lost compared to the UUO conditions, which represents a classic model of tubulointerstitial fibrosis^3^.

Fibrosis is always associated with a chronic inflammatory process controlled by a variety of factors and processes, among which the reciprocal influence of interleukins (ILs), matrix metalloproteinases (MMPs), and tissue inhibitors of matrix metalloproteinases (TIMPs) is of particular interest^41,42^. Normally, the mesangial cells, tubular epithelial cells, and other kidney cells can produce MMP2 and 9 only in small amounts. However, during renal fibrosis, the expression of MMP2 and 9 is rapidly upregulated due to abnormal activation and interaction of multiple cell signaling pathways^43^. MMP signaling usually occurs through the MAPK/ERK 1/2 pathway in the cell, although signaling may also occur through the nuclear factor-kB (NF-kB) pathway, which is known to control the production of inflammatory factors^44,45^. Although MMPs degrade ECM and promote remodeling of the renal connective tissue, they can also contribute to the development of renal fibrosis and ECM accumulation. Indeed, MMP2, highly expressed in the kidney, induced the release of latent TGF-β1^46^ and provoked an epithelial-to-mesenchymal transition^47^. Also, increased expression of both TIMPs have been associated with glomerulosclerosis^48,49^ and ECM accumulation. MMP9 also plays an important role in the initiation and development of CKD^43^. Renal fibrosis is known to be associated with a decrease in MMP9 expression in the cytoplasm of normal tubular cells and increased expression of MMP9 in the nuclei of tubular atrophic renal tubules^50^.

There is limited information on the relationship between proinflammatory cytokines, MMP activity and progesterone signaling. The effects of progesterone on MMP expression and activity have not been studied as extensively as the effects of other sex hormones. There was an evidence that progesterone suppressed MMP production in fibroblasts of the reproductive tract^51^, prevented tissue degradation during the luteal phase of the menstrual cycle^52^, and decreased MMP2 expression and activity in cervical fibroblasts^53^. Probably progesterone does not directly promote or inhibit MMP transcription but instead inhibits the transcription of cytokines, such as IL1, which can lead to decreased MMP transcription^54^. Indeed, progesterone inhibits the production of IL1α и IL1β by mononuclear leukocytes^55^ and Th1-cells^56^. The hypothesis about cytokine-mediated effects of progesterone is supported by the fact that progesterone inhibits the NF-κB pathway associated with the expression of key regulatory genes for immunity and inflammation^56^. Regarding the relationship between ECM remodeling, cytokines, and progesterone in the kidney, we found positive correlations between *MMP2, TIMP1, TIMP2* and *IL1A,* whereas weak correlations or their absence were shown for *IL1B* и *IL18* (see supplementary Fig. S2). Based on the described effects of sex hormones on MMPs^54^, we expected progesterone to decrease MMP2 expression and activity by suppressing *IL1A* and *IL1B*. However, in our experimental model, we found no effect of progesterone on the expression of key pro-inflammatory cytokines: IL1α, IL1β, TNFα, IL18 (Fig. 6).

**Figure 7:**
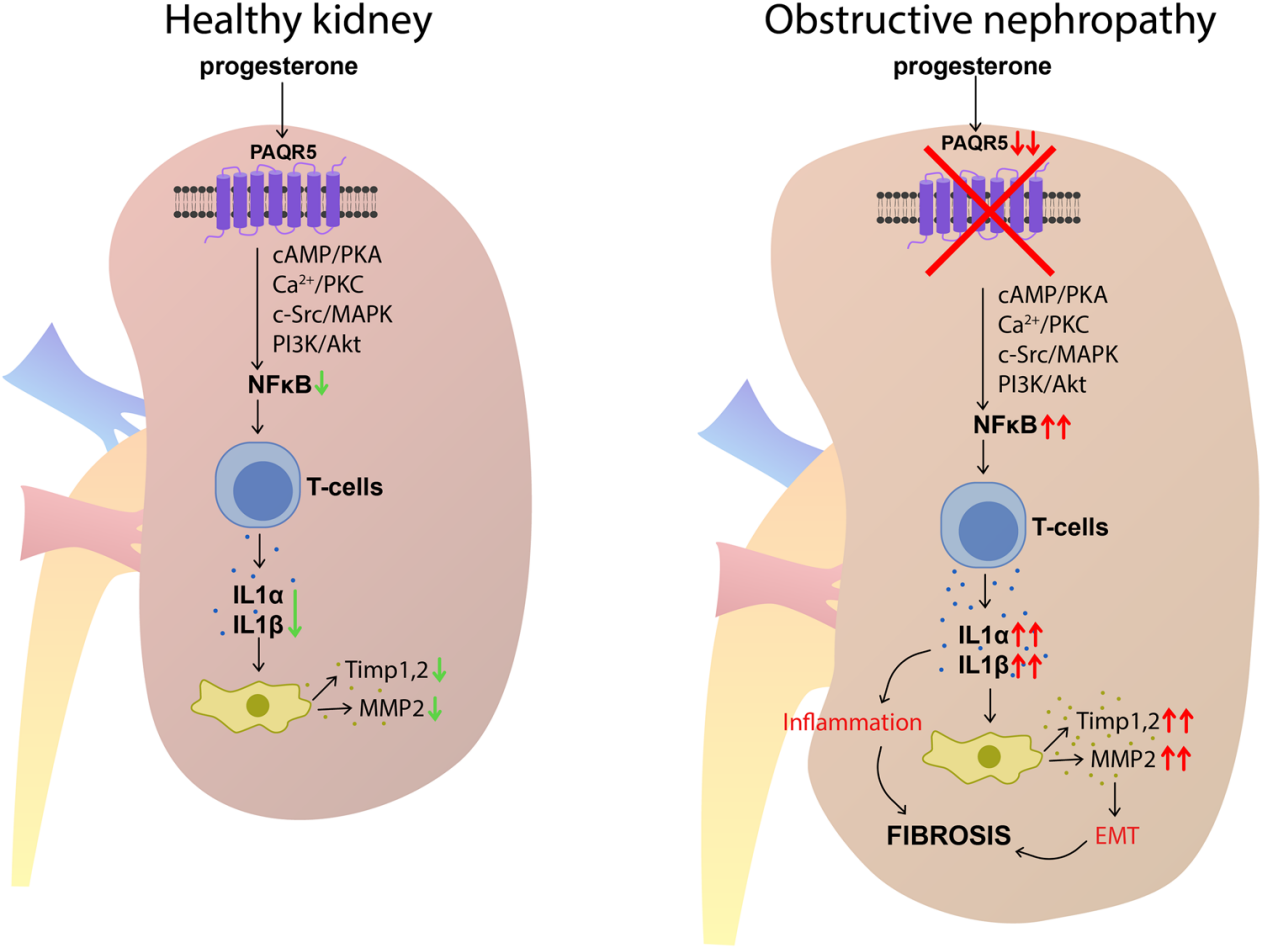
Proposed mechanisms of PAQR5-mediated release of ILs, MMPs and TIMPs in healthy and obstructive kidneys. PAQR5 stimulation in a healthy kidney is accompanied by activation of non-genomic signaling pathways and inhibition of NFkB, leading to suppression of ILs production by T-lymphocytes, and production of MMP2, Timp1 and Timp2 by cells of kidney. In obstructive nephropathy, PAQR5 expression is dramatically decreased, leading to increased NFkB and ILs production. The release of ILs stimulates the activity of MMP2, TIMP1, TIMP2. All these pathological events lead to inflammation, epithelial-to-mesenchymal transition (EMT) and renal fibrosis.

We suggest that the main reason for the lack of progesterone effect on the expression of cytokines and MMP2 as well as on the expression of fibrosis markers is a significantly decreased expression of PAQR5. Based on recent studies, we propose the hypothetical mechanism of PAQR5-mediated release of ILs, MMPs, and TIMPs in a healthy and obstructive kidney (Fig. 7) that could explain our experimental data. Thus, it can be concluded that in a therapy involving the administration of progesterone, it is necessary to know whether the target organ has sufficient levels of the appropriate receptors and whether it is possible to stimulate the expression of these receptors. Furthermore, we assume PAQR5 to be a promising prognostic marker for the kidney fibrosis progression. However, further studies are needed to clarify the dynamics of PAQR5 expression decline in ureteral obstruction or other types of CKD.

## Materials and methods

### Animals

Healthy female Wistar rats 12 weeks of age (250–300 g) were housed under controlled conditions with a 12-h light/dark cycle at 22 ± 2 °C, allowed free access to tap water and were fed with standard rat chow ad libitum. Rats were used according to animal protocols evaluated and approved by the Animal Ethics Committee of the A.N. Belozersky Institute of Physico-Chemical Biology: Protocol 2/20 from February 12, 2020. All procedures were in accordance with Federation of Laboratory Animal Science Associations (FELASA) guidelines and reported in accordance with ARRIVE guidelines.

### Experimental design

Rats were randomly divided into 5 experimental groups: intact rats (N); unilateral ureteral obstruction (UUO, from the left kidney), bilateral ovariectomy (OVX); OVX followed by UUO (OU); OVX followed by UUO with progesterone administration (P). UUO and OVX were performed as described^3,57^. Briefly, for all surgical procedures, rats were anesthetized with isoflurane. For UUO, animals were placed supine on a heating pad and the left ureter was visualized, double ligated with 3–0 silk, and dissected. For OVX, rats were placed prone on a heating pad, and the ovaries were identified and removed after ligation of the uterine horns. For OU rats, ureteral obstruction was performed 2 weeks after ovariectomy. After all surgical procedures a single dose of ciprofloxacin (4 mg/kg) was injected intraperitoneally at the wound site. Rats were sacrificed after 2 weeks of ureteral obstruction. Kidneys were photographed and weighted. The left kidneys (with obstructed ureters) were removed, decapsulated, and cut into pieces. The upper pole of the kidney was placed in 10% buffered formalin, while the other parts of the left kidney were homogenized in an ice-cold RIPA lysis buffer (Millipore, Temecula, CA, USA) containing 1 mM phenylmethylsulfonyl fluoride (Thermo Fisher Scientific, USA) and protease/phosphatase inhibitor cocktail (Cell Signaling Technology, Inc., USA) or PBS buffer for further Western blotting or zymography, respectively. The lower pole of the left or right (contralateral) kidney was taken for further RT-PCR analysis. Protein content in the samples was measured using a commercial kit based on bicinchoninic acid (Sigma, USA). Blood samples were collected for measurement of plasma progesterone levels. The relative kidney weight was estimated as the ratio of kidney weight to body weight expressed as a percentage.

### Progesterone supplementation and plasma progesterone levels

**Figure 8:**
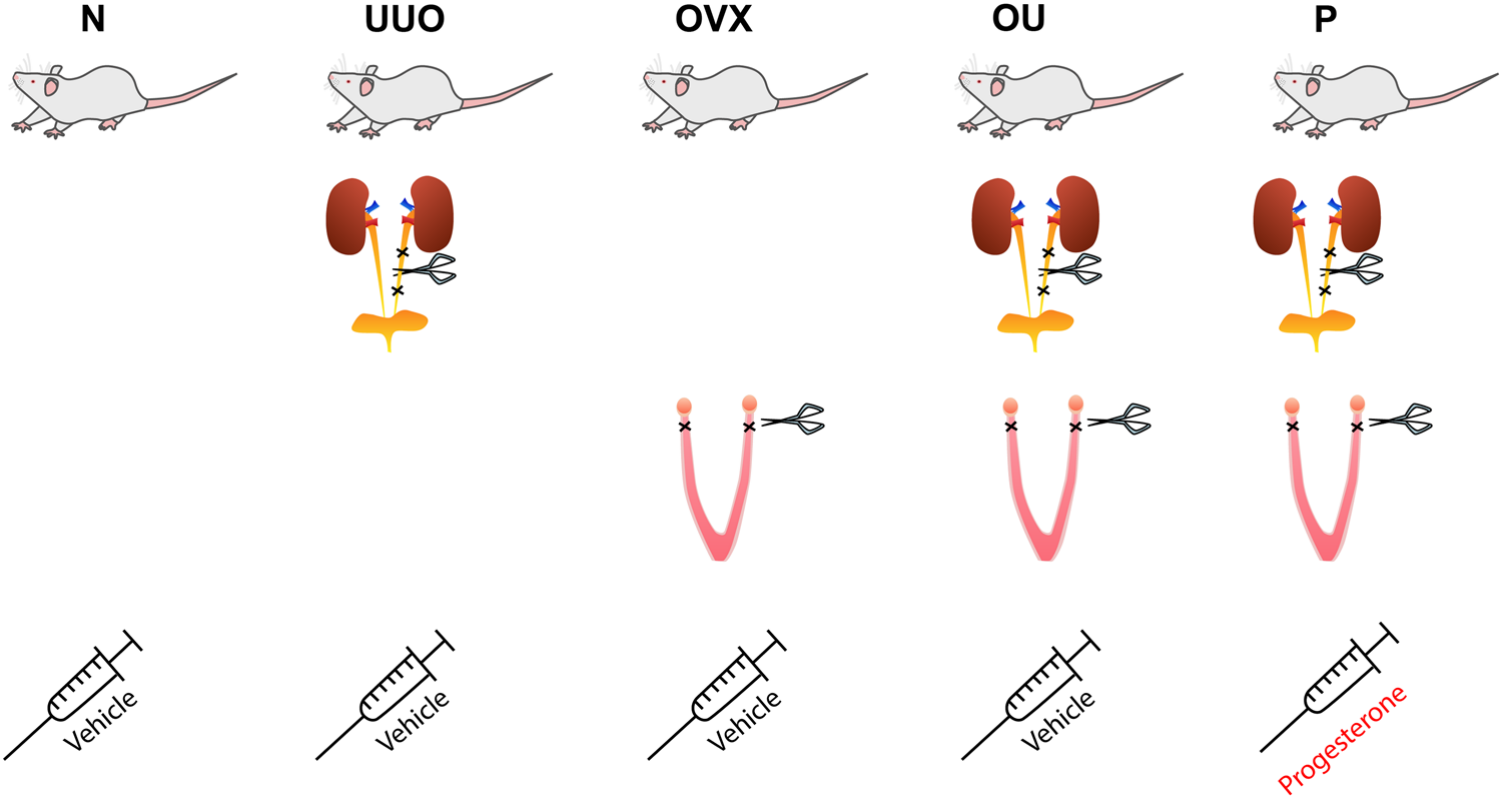
Progesterone supplementation, experimental design.

OU rats receiving progesterone were injected subcutaneously with 10 mg/kg progesterone (P8783, Sigma-Aldrich, Germany) diluted in propylene glycol daily for 2 weeks after OVX. Figure 8 shows the protocol of drug administration and surgery procedures. Animals that did not receive progesterone were injected with propylene glycol only (500 μl/kg). The chosen dose of progesterone was based on various studies^37,58,59^. Plasma progesterone levels were measured using an ELISA kit (Immunotech, Russia).

### Western blotting

Western blotting was performed using the conventional method under denaturing conditions. Samples were loaded onto 10% SDS-PAGE (10, 20, 40 μg total protein per well for α-SMA, MMP9 and PAQR5 detection, respectively) or 20% SDS-PAGE gels (30 μg total protein per well for detection of IL1α and IL18) and transferred to a PVDF membrane (Amersham Pharmacia Biotech, Buckinghamshire, UK) after electrophoretic separation using the Trans Blot Turbo Transfer System (BioRad, USA). Membranes were incubated for 45 min at RT with 5% nonfat milk in TBS containing 0.1% Tween-20 (Helicon, Russia) followed by three washes and then with primary antibodies diluted in 0.1% BSA, against α-SMA 1:1000 (ab5694, Abcam, USA), MMP9 1:1000 (ab38898, Abcam, USA), PAQR5 1:500 (ab79517, Abcam, USA), IL1α 1:1000 (GTX74157, GeneTex, USA), IL18 1:1000 (ab191860, Abcam, USA), and β-actin 1:2000 (9336L, Sigma-Aldrich, USA) at 4 °C overnight. Membranes were washed and incubated with secondary goat anti-rabbit or anti-mouse IgG antibodies conjugated with horseradish peroxidase 1:10,000 (IMTEK, Moscow, Russia) for 60 min at 37 °C. After washing, membranes were incubated for 5 min with Advansta ECL Bright chemiluminescence kit (Advansta, USA) and scanned with ChemiDoc MP Imaging System (BioRad, USA). Band luminescence intensity was quantified using ImageLab 6.0.1 software (BioRad, USA). Band densities were normalized to β-actin.

### Zymography

Homogenized samples were loaded (20 μg total protein per well) onto a 10% SDS-PAGE gel containing 2 mg/ml gelatin (Sigma-Aldrich, Germany). After electrophoresis, the gel was incubated in a renaturation buffer (Invitrogen, Novex, Carlsbad, CA, USA) and activated in a development buffer (Invitrogen, USA). The gel was stained with 0.25% Coomassie Brilliant Blue R-250 for about 1 h, then bleached until gelatinolytic bands were visible. MMP activity, observed as a clear band of digested gelatin on a blue background, was quantified using ImageLab 6.0.1 densitometric image analysis software (Bio-Rad, USA).

### Histological analysis

Paraffin‐embedded, 4 μm sections of renal tissue were stained with Mallory trichrome kit (Biovitrum, St. Petersburg, Russia). Stained kidney slices were observed with Nanozoomer S210 microscope with 20x/NA 0.75 lens, zoom 2x (Hamamatsu, Japan). On each slice, ten images from the cortex were randomly selected for analysis. Slices were examined for fibrosis (area of collagen deposition) in blinded fashion in ImageJ software (NIH, Bethesda, MD, USA).

### RT-PCR

Kidney tissue was homogenized in an appropriate volume of Trizol reagent (Thermo Fisher Scientific Inc., Invitrogen, USA) using Potter homogenizer. After isolation of the aqueous phase containing nucleic acids using chloroform and treatment with 96% ethanol, further steps of total RNA isolation and treatment with DNase were performed on RNeasy plus mini kit (QIAGEN GmbH, Germany) following the protocol recommended by the manufacturer. Following RNA extraction, the concentration and purity were determined by Nanodrop spectrophotometer ND-1000 (Thermo Fisher Scientific, USA). Reverse transcription was performed using the MMLV RT kit (Evrogen, Russia). The RT-PCR was performed on Bio-Rad Real-Time PCR System (Bio-Rad, USA) by using SYBR Green as a double-strand DNA-specific binding dye. Amplification was carried out using 5X qPCRmix-HS mastermix (Evrogen, Russia). Primers (DNA-synthesis, Russia) used in this study (supplementary Table S1) were designed with the Beacon Designer 7 program (Premier Biosoft Int., USA). The primer efficiencies were calculated by generating a standard curve for each target gene using a five-fold serial dilution of the cDNA pool and were in the range of 1.8–2.0. mRNA expression levels were calculated as *E*^−*Ct*^, where *E* is the primer efficiency and *Ct* is the cycle number on which product fluorescence rose above the threshold level. These values were normalized to the geometric mean of the threshold cycles of two housekeeping genes (60S acidic ribosomal protein P0 (*RPLP0*) and hypoxanthine–guanine phosphoribosyltransferase (*HPRT*)), to level individual differences between animals^60^.

### Statistical analysis

Statistical analysis was performed with the GraphPad Prism 8 (GraphPad Software Inc., USA). The data was analyzed by parametric one-way ANOVA with Sidak’s multiple comparison test or nonparametric Kruskall-Wallis test followed by Dunn’s multiple comparison test based on their distribution normality (Shapiro–Wilk normality test). Pearson correlation was used. The results are presented as mean ± SEM with a *p < 0.05, **p < 0.005, ***p < 0.0005, ****p = 0.0001 considered as statistically significant. All groups had N ≥ 3 animals.

### Supplementary Information

Supplementary Information.

## Data Availability

The data that support the findings of this study are available from the corresponding author upon request.

## Abbreviations

CKD: Chronic kidney disease
ECM: Extracellular matrix
IL1α: Interleukin 1 alpha
IL1β: Interleukin 1 beta
ILs: Interleukins
MMP 2: Matrix metalloproteinase 2
mPRγ/PAQR5: Membrane progesterone receptor gamma, or progestin and adipoQ receptor 5
mPRs: Membrane progesterone receptors
nPRs: Nuclear progesterone receptors
OVX: Ovariectomy
TGFβ1: Transforming growth factor β1
TIMP: Tissue inhibitor of matrix metalloproteinase
UUO: Unilateral ureteral obstruction
