# Supplementary material for: Decreased renal expression of PAQR5 is associated with the absence of a nephroprotective effect of progesterone in a rat UUO model
Source: Sci Rep. 2023 Aug 8;13:12871. doi: 10.1038/s41598-023-39848-2 (PMC10409855; doi:10.1038/s41598-023-39848-2)
Supplement: Supplementary file 1 — Supplementary Information. [file 41598_2023_39848_MOESM1_ESM.pdf]

Supplementary information

Decreased renal expression of PAQR5 is associated with the absence of a nephroprotective effect of progesterone in a rat UUO model

Abramicheva P.A.[1], Semenovich D.S.[1], Zorova L.D.[1,2], Pevzner I.B.[1,2], Sokolov I.A.[1,3], Popkov V.A.[1,2,4], Kazakov E.P.[1], Zorov D.B.[1,2], Plotnikov E.Y.[1,2]

[1]*A.N. Belozersky Institute of Physico-Chemical Biology, Lomonosov Moscow State University, Moscow 119234, Russia*

[2]*V.I. Kulakov National Medical Research Center of Obstetrics, Gynecology, and Perinatology, Moscow 117997, Russia*

[3]*Mendeleev University of Chemical Technology of Russia, Moscow 125047, Russia*

[4]*MSU Institute for Artificial Intelligence, Lomonosov Moscow State University, Moscow 119234, Russia*

Correspondence: abramicheva.polina@belozersky.msu.ru (P.A.A.); plotnikov@belozersky.msu.ru (E.Y.P.).

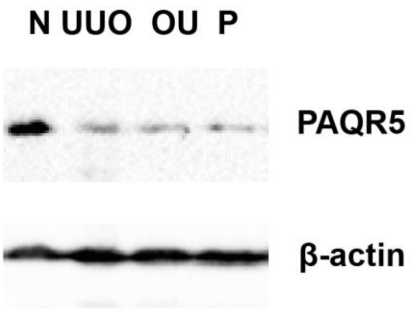

Figure S1. Levels of PAQR5 protein in the UUO-exposed kidney estimated by Western-blotting.

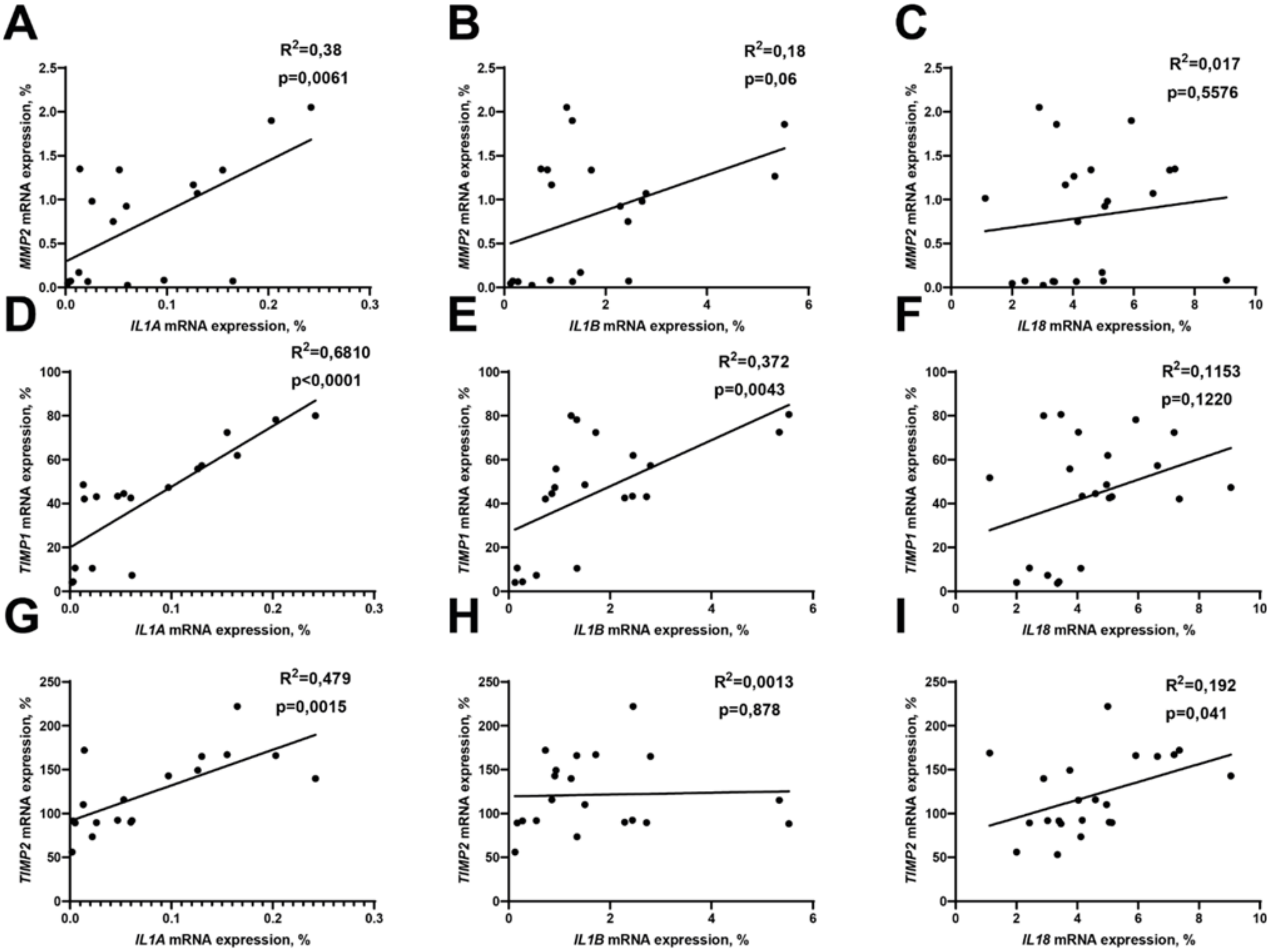

Figure S2. Correlations between interleukins and regulators of ECM accumulation. Scatter plot with linear regression shows a correlation between *MMP2* (A-C), *TIMP1* (D-F), *TIMP2* (G-I) and *IL1A*, *IL1B*, *IL18* mRNA expression in all experimental groups. The Pearson squared correlation coefficient (R^2) and p-value are shown, n=18-22.

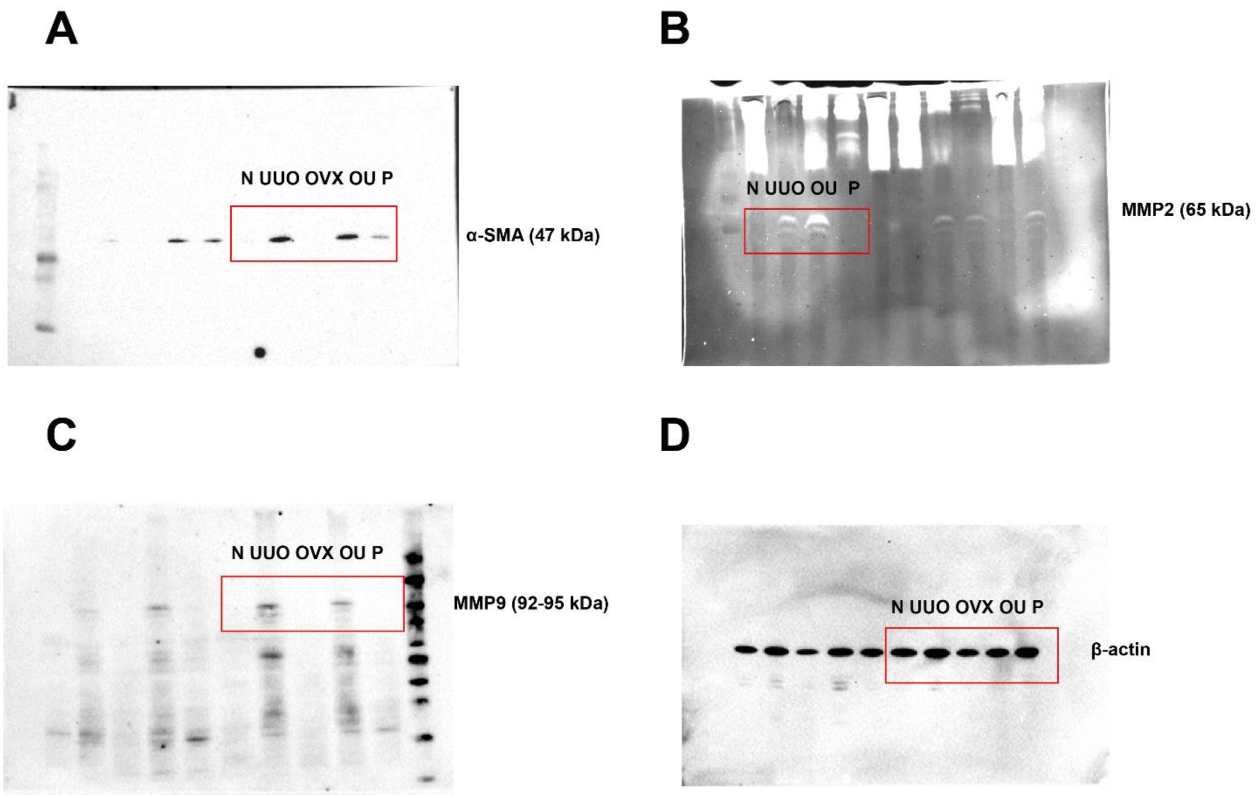

Figure S3. Raw images of western blots and zymogram gel shown in Fig. 4, α-SMA levels (A), MMP2 activity (B), MMP9 levels (C) in the left (exposed) kidney. (D) β-actin as loading control for α-SMA normalization.

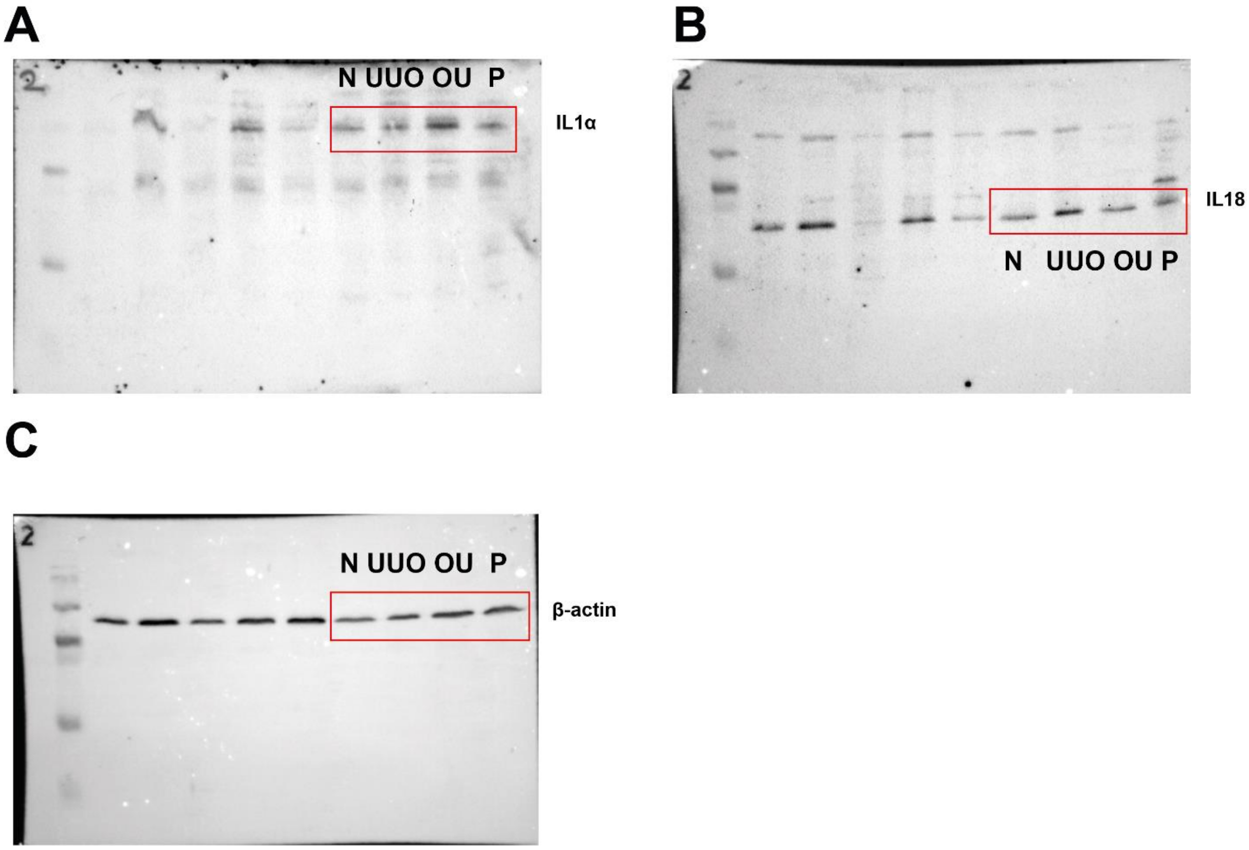

Figure S4. Raw images of western blots shown in Fig. 6, IL1α (A), IL18 (B), β-actin (C) protein.

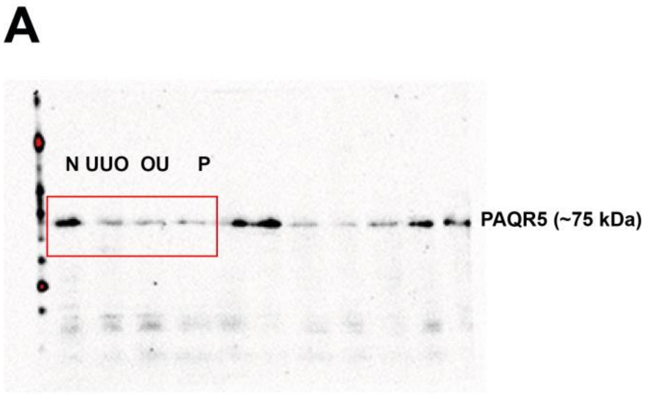

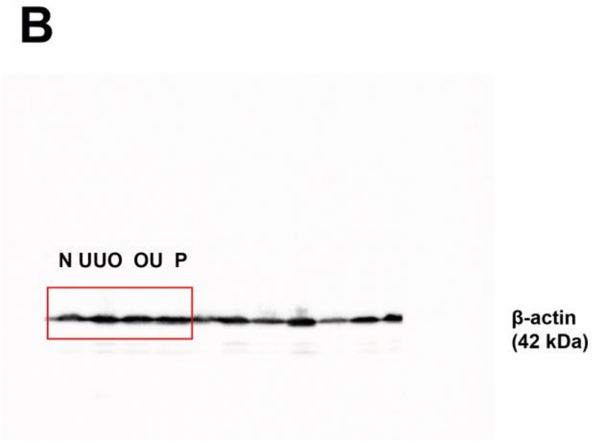

Figure S5. Raw images of western blots shown in Fig. S1, PAQR5 (A), β-actin (B) protein.

Table S1. Sequences of primers used for gene expression estimation.

Protein	Rat gene name	Primer nucleotide sequence (5’- to 3’)	PCR product size, bp
Membrane progesterone receptor γ	*PAQR5* for	ACTATGGTGCGGTCAATCTC	172
	PAQR5 rev	TGGAGTTCAAGAAACCTGGAG	
Nuclear progesterone receptor	*PGR* for	GGAGGTCGTAAGTTTAAGAAGTTC	173
	PGR rev	ATGAGCAGGTTGATGAGTGG	
Transforming growth factor β	*TGFB1* for	CGTACACAGCAGTTCTTCTCT	136
	TGFB1 rev	ATGACATGAACCGACCCTTC	
Fibronectin 1	*FN1* for	TCACAGGGTACAGGATTGT	144
	FN1 rev	TCTCCTCCACAGCATAGATAG	

Matrix metalloproteinase 2	*MMP2* for	GGACAAGAATCAGATCACATACAG	204
	MMP2 rev	TTGCCGTCAAATGGGTATCC	
Collagen type I α1 chain	*COL1A1* for	TCAAGATGGTGGCCGTTACT	166
	COL1A1 rev	CATCTTGAGGTCACGGCATG	
Tissue inhibitor of metalloproteinase 1	*TIMP1* for	TAAAGCCTGTAGCTGTGCCC	160
	TIMP1 rev	AGCGTCGAATCCTTTGAGCA	
Tissue inhibitor of metalloproteinase 2	*TIMP2* for	TAATTGCAGGGAAGGCGGAA	275
	TIMP2 rev	CGCGCAAGAACCATCACTTC	
Tumor necrosis factor α	*TNFA* for	CCTCACACTCAGATCATCTTCTC	281
	TNFA rev	GGCTGACTTTCTCCTGGTATG	

Interleukin 1β	*IL1B* for	ACACAGGACAGGTATAGATTCTTC	119
	IL1B rev	GGACAGAACATAAGCCAACAAG	
Interleukin 1α	*IL1A* for	AACACAGGCTTGTCTTCTCC	291
	IL1A rev	GACCATCCAACCCAGATCAG	
Interleukin 18	*IL18* for	TAGACATCCTTCCATCCTTCAC	169
	IL18 rev	GACCAAGTTCTCTTCGTTGAC	
60S acidic ribosomal protein P0	*RPLP0* for	CACAGTACCTGCTCAGAACAC	138
	RPLP0 rev	ACCTTGTCTCCAGTCTTTATCAG	
Hypoxanthine-guanine phosphoribosyltransferase	*HPRT* for	GCTATAAGTTCTTTGCTGACCTG	149
	HPRT rev	ATCTCCACCAATAACTTTTATGTCC	
